# Attraction by pairwise coherence explains the emergence of ideological sorting

**DOI:** 10.1093/pnasnexus/pgae263

**Published:** 2024-07-08

**Authors:** Federico Zimmerman, Lucía Pedraza, Joaquín Navajas, Pablo Balenzuela

**Affiliations:** Laboratorio de Neurociencia, Universidad Torcuato Di Tella, Av. Figueroa Alcorta 7350, C1428BCW, Buenos Aires, Argentina; Consejo Nacional de Investigaciones Científicas y Técnicas (CONICET), Godoy Cruz 2290, C1425FQB, Buenos Aires, Argentina; Escuela de Negocios, Universidad Torcuato Di Tella, Av. Figueroa Alcorta 7350, C1428BCW, Buenos Aires, Argentina; Departamento de Física, Facultad de Ciencias Exactas y Naturales, Universidad de Buenos Aires, Pabellón 1, Ciudad Universitaria, C1428EGA, Buenos Aires, Argentina; Harvard Business School, Harvard University, Soldiers Field Road, Boston, MA 02163, USA; Digital, Data and Design Institute, Harvard University, Soldiers Field Road, Boston, MA 02163, USA; Departamento de Física, Facultad de Ciencias Exactas y Naturales, Universidad de Buenos Aires, Pabellón 1, Ciudad Universitaria, C1428EGA, Buenos Aires, Argentina; Instituto de Física Interdisciplinaria y Aplicada (INFINA), CONICET, Pabellón 1, Ciudad Universitaria, C1428EGA, Buenos Aires, Argentina; Laboratorio de Neurociencia, Universidad Torcuato Di Tella, Av. Figueroa Alcorta 7350, C1428BCW, Buenos Aires, Argentina; Consejo Nacional de Investigaciones Científicas y Técnicas (CONICET), Godoy Cruz 2290, C1425FQB, Buenos Aires, Argentina; Escuela de Negocios, Universidad Torcuato Di Tella, Av. Figueroa Alcorta 7350, C1428BCW, Buenos Aires, Argentina; Departamento de Física, Facultad de Ciencias Exactas y Naturales, Universidad de Buenos Aires, Pabellón 1, Ciudad Universitaria, C1428EGA, Buenos Aires, Argentina; Instituto de Física Interdisciplinaria y Aplicada (INFINA), CONICET, Pabellón 1, Ciudad Universitaria, C1428EGA, Buenos Aires, Argentina

**Keywords:** political polarization, opinion dynamics, agent-based models, political psychology

## Abstract

Political polarization has become a growing concern in democratic societies, as it drives tribal alignments and erodes civic deliberation among citizens. Given its prevalence across different countries, previous research has sought to understand under which conditions people tend to endorse extreme opinions. However, in polarized contexts, citizens not only adopt more extreme views but also become correlated across issues that are, a priori, seemingly unrelated. This phenomenon, known as “ideological sorting”, has been receiving greater attention in recent years but the micro-level mechanisms underlying its emergence remain poorly understood. Here, we study the conditions under which a social dynamic system is expected to become ideologically sorted as a function of the mechanisms of interaction between its individuals. To this end, we developed and analyzed a multidimensional agent-based model that incorporates two mechanisms: homophily (where people tend to interact with those holding similar opinions) and pairwise-coherence favoritism (where people tend to interact with ingroups holding politically coherent opinions). We numerically integrated the model's master equations that perfectly describe the system's dynamics and found that ideological sorting only emerges in models that include pairwise-coherence favoritism. We then compared the model's outcomes with empirical data from 24,035 opinions across 67 topics and found that pairwise-coherence favoritism is significantly present in datasets that measure political attitudes but absent across topics not considered related to politics. Overall, this work combines theoretical approaches from system dynamics with model-based analyses of empirical data to uncover a potential mechanism underlying the pervasiveness of ideological sorting.

**Significance statement**

We investigate the mechanisms behind ideological sorting, a phenomenon in which people's opinions become aligned on seemingly unrelated topics. By implementing a multidimensional agent-based model that includes only experimentally validated psychological phenomena such as homophily (the tendency for people to interact with those who share similar opinions) and pairwise-coherence favoritism (the tendency for people to interact with ingroups that hold politically coherent opinions), we found that ideological sorting is primarily driven by the latter. Moreover, we support our findings by linking the model to empirical data, revealing that the influence of pairwise-coherence favoritism is present in political attitudes but absent in issues not considered related to politics.

## Introduction

The increasing political polarization (1–3) has become a worrying concern in many different countries (4) and a serious threat to society and democracy itself (5). Polarization drives hatred among family members (6), enables the spread of misinformation (7, 8), promotes the segregation of societies, and reduces the chances of coherently responding to large-scale crises, as recently demonstrated by the COVID-19 pandemic (9–11). Concerned about the risks and societal impacts of this phenomenon, researchers and policymakers have tried to develop interventions to reduce polarization, obtaining mixed results, and demonstrating the complexity of the problem (12–14). In this context, understanding why societies tend to become more polarized has become a crucial issue in the behavioral and social sciences.

One promising way to understand the emergence of political polarization is by studying the behavior of agent-based models (ABMs) under different conditions of social influence and interactions. From a modeling point of view, several mechanisms have been explored in order to explain issue polarization. For instance, bounded confidence (15–18), negative influence or repulsion (19–21), or homophily in conjunction with other mechanisms such as social influence in cultural vectors (22–25), persuasive arguments theory (26, 27), or biased assimilation (28). Based on a few simplistic assumptions, most studies have focused on understanding under which conditions individuals may polarize and become more extreme on one single topic (29), even though this simplification lacks the possibility of modeling phenomena that arise due to the interplay of multiple issues.

While disagreement on policy issues has been extensively studied in previous research, relatively less attention has been paid to understanding why people tend to be more aligned across diverse and seemingly unrelated topics (2, 30, 31). For example, an individual who supports the women's right to voluntarily terminate pregnancy will be more likely to support stricter legislation on gun control, even though these topics are, a priori, unrelated to each other. While there is consensus on the existence and importance of this phenomenon, known as “ideological sorting”, there is debate about whether it has been increasing in recent years (32–35). In any case, the micro-level mechanisms underlying the emergence of ideological sorting in social systems remain poorly understood.

In a recent paper, a set of large-scale behavioral experiments have shown that people not only hold politically coherent opinions across very different issues but also that this property, i.e. political coherence, increases interpersonal attraction among co-partisans (36). In other words, individuals who hold coherent opinions are more attractive than those individuals having some degree of ambivalence in their attitudes (e.g. a person who is anti-abortion but supports gun control). This idea is in line with previous findings showing that people favor pro-norm deviants (37, 38). However, whether and how this driver of interpersonal attraction, called “pairwise-coherence favoritism”, relates to macro-level patterns of political polarization and partisan-ideological sorting remains largely unknown.

This overreaching aim necessarily requires the formulation of multidimensional dynamic models where polarization could arise in independent individual topics (with no correlation between them) or as ideological states where topics are aligned and correlations between them are pronounced. In proportion, there are far fewer models that study opinion in multidimensional spaces, which could give rise to these phenomena (39–44). Ideological sorting has been previously modeled by considering continuous opinions and nonorthogonal and overlapping topics (45) or directional voting (46). However, none of these approaches have tested the effect of pairwise-coherence favoritism, given that it is a recently uncovered empirical finding in the social sciences. Here we show that by incorporating this rule of interpersonal attraction, issue alignment emerges, even when agents start from a random distribution of opinions. To demonstrate this, we first formulated the model, numerically integrated its master equations, and ran multiple computational simulations, always obtaining the same consistent results. We then compared different final states with actual data from multiple datasets that include 24,035 opinions on different controversial issues. All analyses indicate that homophily alone is insufficient to account for ideologically sorted states, highlighting the significance and impact of pairwise-coherence favoritism in political interactions.

## The model

In previous research (36), the authors performed three different studies in different countries and found that people are more attracted to politically coherent ingroups rather than to those who hold ambiguous or ambivalent opinions. In that study, someone was considered coherent if her/his opinions were aligned with her/his political ideology. For example, a coherent Democrat would be pro-choice regarding abortion and also favor stricter gun control. In two live crowd experiments, participants were arranged in dyads, discussed five controversial topics, and completed an interpersonal attraction questionnaire. In both cases, interpersonal liking increased as a function of similarity, but also of pairwise coherence. Interpersonal liking was found to be nonreciprocal: people with ambivalent and uncertain political views were more attracted to coherent ingroups than vice versa. These results were validated by performing an online preregistered experiment where political coherence was experimentally manipulated. Overall, these empirical results suggested that liking in the political domain may not be solely driven by homophily but by more complex notions of group affiliation such as pairwise coherence.

### Agents, opinions, and communities

We considered a system of N agents. Each agent holds a multidimensional vector opinion, where each dimension stands for the agent's opinion on a particular issue (for the sake of simplicity, we considered only two issues). For each issue, the agent could be against, undecided, or in favor of the issue. Two considerations about how opinions are modeled are to be made: Firstly, the inclusion of a neutral or undecided state is grounded on modeling approaches (47–50), behavioral experiments (48, 51), and surveys (e.g. ANES). Secondly, in order to define a metric for quantifying pairwise coherence, we assumed that every political issue expressing a right-wing opinion will be labeled as +1, a left-wing opinion as −1, and undecided as 0. Additionally, we explored the implications of agents having more than three possible opinions. For example, when considering a scenario with five possible opinions, we found that the results are the same as when considering only three possible opinions, though the model becomes more complex (see supplementary material appendix for details).

**Fig. 1.**
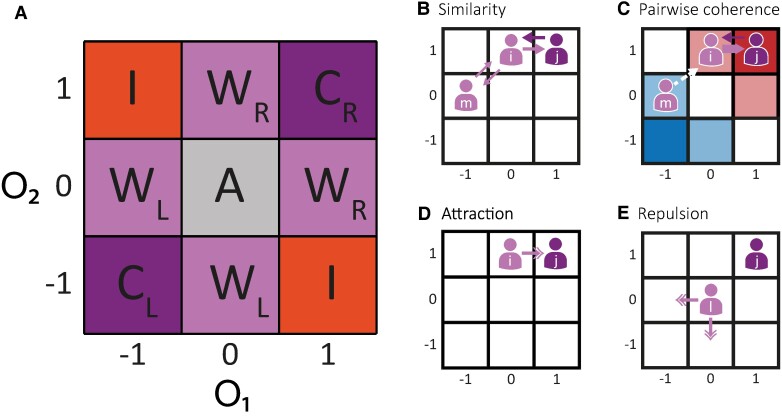
The model. **A)** Each agent holds an independent opinion on two different topics and, according to their opinions, they are classified in four different communities: coherent (*C*), incoherent (*I*), weak (*W*), or apathetic (*A*). Additionally, agents' opinions define their ideologies. Agents can be considered left- or right-wing (L or R) depending on whether most of their opinions are left- or right-wing oriented. **B)** Similarity measures how similar the opinions between two agents are. It is a commutative measure. Similarity between agents *i* and *j* (*S_ij_* = *S_ji_* = 0.75) is higher than similarity between *i* and *m* (*S_im_* = 0.5) as the Manhattan distance is lower. **C)** Pairwise coherence measures the target agent's political coherence. This is a noncommutative measure. *C_ji_* is 1 because agent *j* is coherent, but *C_ij_* is 0.5 because *i* is a weak agent. *C_mi_* is 0 because the agents do not share the same ideology. While *i* is a right-wing oriented agent, *m* is left-wing oriented. **D)** Interactions between similar agents are attractive (*S* ≥ *T*). After an interaction between *i* and *j*, *i* changes its opinion and moves closer to *j*. **E)** Interactions between dissimilar agents are repulsive (*S* < *T*). After an interaction between l and *j*, l changes its opinion and moves further from *j*. This movement could be to the left or down with probability 0.5.

Therefore, in terms of coherence, we can define four different communities: Coherent agents (*C*): Agents that hold assertive and matching opinions on both issues. Left-wing coherent agents (*C_L_*) hold both left-wing opinions (*O* = (−1, −1)) and right-wing coherent agents (*C_R_*) hold right-wing opinions (*O* = (1,1)). Incoherent agents (*I*): Agents that hold assertive but opposite opinions on the two different issues. Incoherent agents hold one left-wing opinion and one right-wing opinion (*O* = (−1,1) or *O* = (1, −1)). Weak agents (*W*): Agents that hold an assertive opinion on one issue and are undecided regarding the other one. The agent's ideology is determined by the political leaning of its assertive opinion (*O* = (−1,0) or *O* = (0, −1) are considered left-wing weak agents and *O* = (1,0) or *O* = (0,1) right-wing weak agents). Apathetic agents (*A*): Agents that are undecided on both issues (*O* = (0,0)). Following these definitions, every possible agent's opinions can be mapped onto a 3 × 3 board as shown in Fig. 1A.

In this work, we focused on the populations' dynamics of each community. The agents' opinions were initially independent and uniformly distributed. Because the number of possible combinations of opinions is not the same for each community, the initial proportion of agents for each community varies. For example, the initial proportion of coherent and incoherent agents is 2/9, while the proportion of weak agents is 4/9.

### Definitions of similarity and pairwise coherence

In what follows, we show how we defined similarity and pairwise coherence and how to implement them in the interaction mechanism between agents. First, we defined similarity between the agents *i* and *j* (*S_ij_*) as a function of the Manhattan distance between the agents' opinions (i.e. the sum of the absolute differences of their Cartesian coordinates). If two agents hold the same two opinions, similarity is 1, and it is 0 if they have opposite stances on both issues. This is computed as:


(1) $$S_{ij}=1-\frac{{\left|\left(O_{i}-O_{j}\right)\right|}_{1}}{4}.$$

For example, as depicted in Fig. 1B, for agents *i* and *j* with opinions *O*_i_ = (0,1) and *O*_j_ = (1,1), the similarity between them is *S_ij_* = *S_ji_* = 3/4, while for agent *m* with opinion *O*_k_ = (−1,0), the similarity with *i* is *S_ik_* = *S_ki_* = 2/4.

Second, in order to implement attraction from pairwise coherence, we define two metrics. The agents' ideology is computed as:


(2) $$I_{i}=\frac{O_{i}^{\left(x\right)}+O_{i}^{\left(y\right)}}{2},$$

which ranges from −1 to 1. *I_i_* = −1 corresponds to left-wing coherent agents holding two left-wing opinions and *I_i_* = +1 corresponds to right-wing ones. Weak agents have an absolute value of *I_i_* = 0.5 and incoherent agents *I_i_* = 0. The ideology's sign value corresponds to the agent's leaning: a positive value describes right-wing agents and a negative one describes left-wing agents. Agents whose ideology is 0 are neutral as they do not belong to any of the two groups. Pairwise coherence is computed as:


(3) $$C_{ij}=|I_{i}|\delta_{ij}$$

(where δ is 1 if *i* and *j* have the same ideology's sign and 0 otherwise), depends on both agents' ideology and the partner agent's coherence. It takes positive values for dyads who share the same leaning and is 0 otherwise. Its maximum possible value corresponds to coherent ingroup agents and it is 0 for outgroup agents. This measure is not commutative and this is in line with experimental results that showed that social influence is not always reciprocal (52).

In Fig. 1C, we show an example involving agents *i*, *j,* and *m.* The pairwise coherence that *j* perceives from *i* is *C_ij_* = 0.5, while the coherence that *i* perceives from *j* is *C_ji_* = 1. Meanwhile, the pairwise coherence between *i* and *m* is 0 as they have ideologies with opposite signs. Interestingly, pairwise coherence for neutral agents is 0 with all communities.

### Interactions dynamics

In this model, agents interact with each other and these interactions influence agents' opinions. At each time step, two agents are randomly selected and their interaction would lead one of the agents to influence the other with probability “*P*”. We incorporated two different mechanisms that impact agents' influence: homophily (53, 54) and pairwise coherence (36, 55), as defined previously. This probability of influence is implemented as a linear combination of both mechanisms:


(4) $$P_{ij}=(1-k)S_{ij}+kC_{ij}.$$

The parameter *k* modulates the strength of each of the two terms and ranges from 0 to 1. When *k* is 0, interactions are only driven by homophily and, when it is 1, only by pairwise coherence. Any intermediate value takes into consideration both dynamics as proposed by previous experimental work.

Pairwise interactions lead to opinion changes that could occur only in one of the two issues. Following field-experiment results that showed that partisans who are taken out of their Twitter's echo-chamber became more extreme (56), this model's interactions can be attractive, repulsive, or have no effect, depending on agents' similarity. When agents are similar enough, influence will be attractive and agent *i* will move closer to *j* by changing one of its own opinions as depicted in Fig. 1D. Conversely, for dissimilar agents they can repel or ignore each other. In the case where they are ignored (nonrepulsive model), the model can be thought of as an extension to 2D of a bounded confidence mechanism (15, 18, 57). In the repulsion case, agent *i* changes one of its opinions (selected at random) moving further from agent *j* and reducing their similarity as shown in Fig. 1E. It can happen that the agent cannot move any further in the selected direction and, in this particular case, no change will be made and the interaction would have no effect at all. For example, in the case of an agent holding *O* = (−1, −1) that cannot move further to negative values in any dimension. We set the attraction-similarity boundary, *T*, at similarity *T* = 3/4. This means that for greater or equal similarity values of 3/4, interactions are attractive and they are repulsive or have no effect otherwise. By setting *T* = 3/4, we ensured that there are no attractive interactions between agents from different leanings, thus avoiding contradictions with previous experimental findings (56). Moreover, we explored different values of *T* and found that alternative settings lead to final states where all agents converge to one type of population, a scenario that is not observed in actual opinion data (see supplementary material appendix for details).

### Summary of interaction rules

In summary, let *N* agents hold two different opinions initially randomly distributed. For each time step: (i) Two agents *i* and *j* are randomly chosen and we compute *S_ij_*, *I_ij_*, and *P_ij_* according to their opinions. (ii) Agent *i* influences *j* with probability *P_ij_*. The influence can be attractive, repulsive, or have no effect, depending on the agents' similarity. (iii) The agent *i* can modify its opinion on one of the two issues.

## Master equations

We developed a set of master equations that describe the dynamics of the agents' communities. As at each interaction two agents are randomly selected, the probability of choosing an agent from a particular community depends on the community's proportion. We computed the likelihood of changing opinions after pairwise interactions and obtained the flux's expected values between populations. For example, the probability of a weak agent becoming coherent or vice versa was calculated by considering the four possible scenarios in which this could occur:

- A weak agent interacts with a coherent neighbor with probability WC/2 (where W and C are the proportions of Weak and Coherent agents, respectively). With influence probability given by $P=(1-k)\frac{3}{4}+k$, the weak agent would move to the coherent population.
- A weak agent interacts with another weak agent with an opposite ideology with probability W^2^/4. With probability $P=(1-k)\frac{1}{2}$, it would become coherent due to a repulsive interaction.
- A weak agent interacts with an opposite ideology coherent agent with probability WC/2. With probability $P=(1-k)\frac{1}{4}$, it would move to the coherent population due to a repulsive interaction.
- A coherent agent interacts with an adjacent weak agent with probability WC/2. With probability $P=(1-k)\frac{3}{4}+\frac{k}{2}$, the coherent agent would move to the weak population.

By repeating this analysis with the other communities, we obtained the following equations:


(5) $$\frac{dC}{dt}\;=WC\left(\frac{1-k}{16}+\frac{k}{4}\right)+W^{2}\frac{1-k}{16}$$

(6) $$\frac{dI}{dt}\;=WI\frac{1-k}{16}+W^{2}\frac{1-k}{16}$$

(7) $$\frac{dA}{dt}\;=-AC\frac{1-k}{2}-AI\frac{1-k}{2}$$

(8) $$\frac{dW}{dt}\;=-WC\left(\frac{1-k}{16}+\frac{k}{4}\right)-WI\frac{1-k}{16}-W^{2}\frac{1-k}{8}+AC\frac{1-k}{2}+AI\frac{1-k}{2}$$

**Fig. 2.**
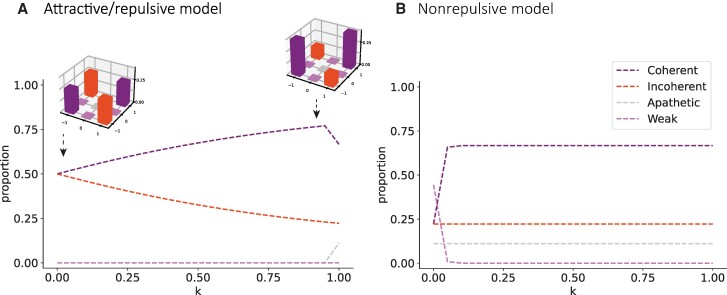
Final states. The model's final states are shown for different values of *k*. **A)** Attractive–repulsive model: The figure depicts the numerical solution of the model. The coherent community is shown in purple, the incoherent one in orange, the apathetic community in gray, and the weak one in pink. For *k* < 1, as *k* increases, so does the final proportion of coherent agents. The top-left inset shows the agents' opinions' mean final distribution for *k* = 0 and the top-right inset for *k* = 0.9. **B)** Nonrepulsive model: The figure depicts the numerical solution of the nonrepulsive model. The coherent community is shown in purple, the incoherent one in orange, the apathetic community in gray, and the weak one in pink. For *k* > 0, the final proportions of coherent agents do not vary significantly with *k*.

We numerically integrate these equations with agents' initial opinions randomly distributed (i.e. *C*(0)=*I*(0) = 2/9, *A*(0) = 1/9, and *W*(0) = 4/9). Then, we observed the final distribution of each community for different values of *k*, as presented in Fig. 2A. Particularly, when *k* = 0, interactions are only driven by homophily. Coherent and incoherent populations exhibit the same behavior (*dC/dt* = *dI/dt*) and, consequently, the final agents' proportions are the same for both communities (Fig. 2A top-left inset). We observe that as *k* increases so does the number of coherent agents (Fig. 2A top-right inset). When *k* = 1, interactions are only driven by pairwise coherence, and, on average, apathetic and incoherent populations do not change over time (*dA/dt* = *dI/dt* = 0).

We conducted an analysis of fixed points and stability for the system of equations. To do that, we set the equations equal to 0 and assumed that *k*≠1. If *W*≠0, from equation 6, we obtained that *W* = −*I*. Given that the four variables represent proportions, their values should be between 0 and 1, it is not feasible for *W* to be nonzero; therefore, *W* = 0. If *A*≠0, from equation 7, we obtained that *C* = −*I*. Since these variables cannot take negative values, *C* and *I* must also be zero. Then, considering that *C* + *I* + *A* + *W* = 1, we concluded that *A* = 1, obtaining a fixed point at *C* = *I* = *W* = 0 and *A* = 1. If *A* = 0, all the equations become null, so the set of points of the form *I* = 1−*C*, *W* = *A* = 0 are also fixed points. To analyze the stability at these fixed points, we linearized the system and calculated the eigenvalues of the Jacobian matrix. The Jacobian for the first fixed point is:


(9) $$\left(\begin{array}{cccc} 0 & 0 & 0 & 0\\ 0 & 0 & 0 & 0\\ -\frac{1-k}{2} & -\frac{1-k}{2} & 0 & 0\\ \frac{1-k}{2} & \frac{1-k}{2} & 0 & 0 \end{array}\right)$$

Thus, the obtained eigenvalues are 0, and the stability of this point cannot be determined. However, we observed that in the linearized system, and near this point, the derivative of *A* remains negative, indicating that the system is likely to be unstable. By examining the eigenvalues of the second set of fixed points, we found that two eigenvalues are 0, one is (−1 + *k*)/2, and another is (−1 + *k*)/16 − *Ck*/4. Therefore, all the eigenvalues are zero or negative, and we only observe attractive behaviors. In summary, we found an unstable fixed point where all agents are apathetic (*A* = 1, *C* = *I* = *W* = 0) and a subspace of stable fixed points given by *W* = *A* = 0.

We also developed and solved the equations for the nonrepulsive variant of the model. The terms involving repulsive interaction are not present and the dynamic equations are as follows:


(10) $$\frac{dC}{dt}=WC\frac{k}{4}$$

(11) $$\frac{dI}{dt}=0$$

(12) $$\frac{dA}{dt}=0$$

(13) $$\frac{dW}{dt}=-WC\frac{k}{4}$$

In this variant, incoherent and apathetic communities, on average, do not change over time. When *k* > 0, all weak agents become coherent, and, as a consequence, their communities' final proportions of agents do not depend on *k*. Figure 2B shows the numerical solutions for the nonrepulsive model. Additionally, we performed computational simulations for both variants of the model and obtained the same results (see supplementary material appendix for details).

## Comparison with empirical data

Here, we focus on one of the multiple phenomena of political polarization: ideological sorting, in which opinions on seemingly unrelated topics display a strong correlation with each other. In the context of the proposed model, higher ideological sorting corresponds to an increase in the proportion of coherent agents. Next, we analyze the extent to which actual opinions on a wide variety of controversial issues are sorted and can be analyzed in the context of the developed model to study the role of homophily and pairwise coherence in opinion dynamics. We work with multiple data sources with 24,035 responses on 67 different polarizing topics. All responses indicate whether participants agree or not to each different issue (Table 1).

**Table 1.** A summary of the most relevant characteristics of the six data sources considered in this work: the source, the year in which the survey was conducted, the number of selected issues, the number of participants who completed each survey, the country, and whether topics were considered related to politics or not. These data sources include 24,035 responses on 67 different topics.

Source and year	Number of selected issues	Number of participants	Country	Topic
Zimmerman et al. 2022	21	5,218	Argentina	Political & nonpolitical
ANES 2020	14	8,280	USA	Political
ANES 2016	28	4,270	USA	Political
Freira et al. 2021	8	1,976	Argentina, Brazil, Uruguay & USA	Political
Pew Research 2020	5	1,013	USA	Political
Pew Research 2014	5	3,278	USA	Nonpolitical

### Data sources

**Zimmerman et al. 2022**. They conducted multiple behavioral experiments in order to understand how people perceive politically coherent individuals. First, they performed an online survey asking 180 participants from Argentina for their opinion on 28 different topics to select relevant and controversial political issues. This survey allowed them to select the five political issues and the five nonpolitical topics they used in an experiment with 5,038 participants.

**ANES 2020 and 2016**. The American National Election Studies (ANES) are nationally representative surveys of eligible American voters. Surveys have been conducted before and after every presidential election since 1948. All the questions are related to US politics. We only used the survey's questions that express participants' approval or disapproval of controversial issues, and we worked with data from the last two surveys. The survey performed before the 2020 presidential election was completed by 8,280 citizens, while the one before the 2016 election by 4,270.

**Freira et al. 2021**. They performed an online behavioral experiment in 2020 in four different countries to understand how partisan differences influenced the COVID-19 pandemic perception. 1,995 participants from Argentina, Brazil, Uruguay, and the USA expressed their opinion on eight different pandemic preventive political policies.

**Pew Research 2020 and 2014 surveys**. The Pew Research Center is an American think tank that provides information on social issues and public opinion trends. Their different representative surveys cover a wide variety of topics, such as US politics, climate, religion, and driverless vehicles. For our purposes, we selected two surveys: one oriented to understand the public's opinion on American federal agencies and the other to study how religion influences the daily lives of Americans. The first one was performed in 2020 and 1,013 participants completed an omnibus survey expressing whether they favor or not different American federal organizations. In the 2014 trends panel survey, 3,278 Americans completed a self-administered web survey covering a wide range of topics including religion and personal opinions. Within this survey, we focused on personal opinions on topics such as lying or meditation.

Although every popular opinion is of interest to political science, we observed that some of the selected datasets cover topics that are part of specific partisan agendas, e.g. abortion or gun control, while others do not relate to any political platform, e.g. pets or food preferences. To confirm that our datasets included both political and nonpolitical issues and to determine whether the distinction between these two categories is not arbitrary, we conducted a preregistered online survey. In this survey, participants on Prolific were asked to what extent they consider the statements from each dataset to be related to politics on a 7-point Likert scale ranging from “not at all related” to “extremely related”. Each participant (N = 100 US citizens, 50 male/female, mean age: 38.8, SD: 12.6) was presented with five statements randomly chosen from six different data sources: two datasets from Zimmerman et al. 2022, ANES 2020 or ANES 2016, Freira et al. 2021, Pew Research 2020, and Pew Research 2014 (Table 1). Participants were instructed to evaluate the set of statements as a whole rather than the individual statements within it. The study received approval from the ethics committee for scientific and technological research at the Universidad Abierta Interamericana (protocol number 0-1104), and informed consent was obtained from all participants. Consistent with the preregistration, participants rated the datasets we had considered to be related to politics higher (*M* = 5.66 ± 0.08) compared to the nonpolitical ones (*M* = 1.30 ± 0.06; linear mixed-effects model: *b* = 4.4 ± 0.1, *t*(499) = 39.7, *p* = 2 × 10^−156^). This result supports a consensus-based distinction between political and nonpolitical issues (see supplementary material appendix for details).

### Sorting

For each dataset, we selected the questions that express participants' opinions on a particular topic. Moreover, we only considered controversial issues in which there is not a majoritarian opinion. The selected issues' responses exhibit high variance following our selection criteria (*σ*^2^ > 0.5). Following the proposed model, all responses were mapped into three possible answers: −1, 0, or 1, where 0 expresses an undecided or neutral posture. For some questions this procedure was trivial, but for others, we merged different degrees of acceptance into one unique alternative. For example, all the following answers were considered as an agreement in our model: “agree strongly”, “agree somewhat”, “very favorable”, and “mostly favorable”. This approach was applied to the responses from the ANES data. However, to ensure that our results were not influenced by this classification method, we created two datasets for each ANES survey (2016 and 2020): one following the described merging procedure and another dataset that includes only answers to questions where participants had only three possible responses, thus eliminating the need to collapse data. This approach allows us to control for the influence of response categorization on our findings (see supplementary material appendix for details). Furthermore, in order to observe ideological alignment, we needed to code responses as in the model: right-wing opinions as 1 and left-wing opinions as −1 regardless of how the question was presented. To do so, we followed the procedure proposed by Zimmerman et al. 2022 where they projected the agree/disagree opinions to the data's first principal component, which, in the political domain, is equivalent to coding them as left/right-wing answers and align the responses into their corresponding ideology.

Once we had responses on controversial issues categorized into three possible opinions (typical left-wing, neutral, and typical right-wing), we proceeded to contrast the data's partisan-ideological sorting with the proposed two-dimensional model. This analysis focused on the relationship between the proportion of coherent (*C*) and incoherent (*I*) opinions. For each dataset, we examined every possible pair of opinions to identify the number of participants presenting coherent opinions (both opinions aligning as 1,1 or −1, −1) as opposed to incoherent opinions (1, −1 or −1,1). For each dataset, we calculated a single sorting value for each pair of opinions, which is the proportion of participants expressing coherent opinions relative to the total number of both coherent and incoherent opinions. Then, we computed the average sorting value for each dataset by considering all possible topic pair combinations. Therefore, sorting (*S*) was determined by averaging the proportion of pairwise coherent opinions in relation to the combined total of coherent and incoherent opinions across all potential opinion pairs within each dataset (see supplementary material appendix for details).


(14) $$S=\frac{C}{\left(C+I\right)}$$

**Fig. 3.**
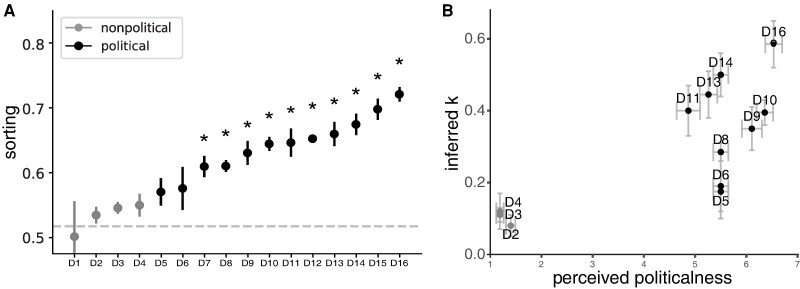
Sorting patterns, model's parameter *k*, and perceived politicalness. **A)** The figure shows the mean sorting value and its standard error (SEM) for each dataset ranked from lowest to highest. Dataset ID numbers correspond to different sets of questions, as detailed in Table S1. Nonpolitical datasets are shown in light gray and political datasets in black. Asterisks indicate whether the sorting value is significantly higher than the one observed in homophilic-only simulations (*P < 0.01). **B)** The figure shows the perceived politicalness obtained from the online survey (*x*-axis), and the model's parameter *k* obtained from the opinions in each dataset through the model (*y*-axis). The figure shows the mean and SEM of these variables for each dataset. Nonpolitical datasets are shown in light gray and political datasets in black.

Figure 3A shows the mean sorting value for each dataset ranked from lowest to highest. Nonpolitical datasets are shown in light gray and political datasets in black. Noteworthy, the political datasets exhibit the highest sorting values.

Furthermore, we followed the same procedure to compute the mean sorting value of 100 simulations where *k* = 0, these are homophilic-only simulations. First, for each simulation's final state, we projected the opinions to their first principal component, and then we computed the corresponding sorting value. The mean sorting value of the simulations (*y* = 0.52) is depicted in a dashed horizontal gray line (Fig. 3A). We observed that none of the nonpolitical sorting values are significantly different from homophilic-only simulations (one sample *t*-test: *t*s < 3.1, *p*s > 0.013) and 10 out of 12 political sorting values are (*t*s > 5.7, *p*s < 0.0004).

Additionally, having the model's numerical solution allowed us to obtain the model's *k*-value that results in a final state matching the sorting value observed in each dataset. Because the proportion of the coherent community, and therefore sorting, increases monotonically with respect to *k* for *k* < 1, we were able to map each sorting value to a corresponding *k*-value. For each dataset, we used the range provided by the standard error of the mean sorting to determine the minimum and maximum *k*-values, and then we computed the mean *k*-value. This parameter allows us to quantify an underlying social mechanism derived from observed opinion data. The value of *k* quantifies the relevance of pairwise-coherence favoritism in relation to homophily across various contexts, such as different issues, countries, or populations. A *k*-value of 0 would indicate that the observed data could be explained solely by considering homophily. A value of 0.5 would highlight that pairwise-coherence favoritism is as relevant as homophilic interactions. A *k*-value of 1 does not take into account the influence of homophily, which contradicts well-established findings in social science. Therefore, we focused our analysis on cases where *k* < 1. For each dataset, we derived a *k*-value from the model that could explain the observed levels of sorting. Moreover, through the online survey, we obtained their perceived politicalness. The results of social experiments have shown that pairwise-coherence favoritism is present in political discussions but not in nonpolitical issues (36). Consistent with this finding, we expected higher *k*-values among datasets categorized as political. As preregistered, we found that the datasets' *k*-values were correlated with their mean perceived politicalness reported in the online survey (Spearman correlation: *r* = 0.62, *p* = 0.03; Fig. 3B).

Taken altogether, by combining the model with actual data, we found that nonpolitical controversial opinions exhibit the lowest observed levels of sorting, which can be explained by homophily and attractive–repulsive interactions. But, in the political domain, homophily alone cannot explain the emergence of the observed levels of ideological sorting. These results are in accordance with recent experimental work that showed that both homophily and pairwise-coherence favoritism are relevant in pairwise political interactions. Moreover, our model allowed us to study how these mechanisms impact the macroscopic opinion landscape. Particularly, how they relate to issue polarization and ideological alignment.

## Discussion

In this work, we considered a multidimensional agent-based model to study how pairwise-coherence favoritism, a finding from experimental psychology, could explain the emergence of ideological sorting. This model considers two well-known and studied phenomena: agents who share similar opinions are more likely to interact and these pairwise interactions can be attractive or repulsive depending on their similarity. Moreover, we included a third and recent finding in which agents are more attracted to coherent ingroups rather than incoherent or outgroup members. Interestingly, by incorporating this last assumption, opinions become more aligned, and we were able to reproduce different correlation patterns similar to those widely observed among political opinions, i.e. ideological sorting.

We developed a two-dimensional model as our main interest was to study the effect of pairwise-coherence favoritism in political interactions and its impact on ideological sorting. The commonly used one-dimensional models of opinion formation lack the possibility to model these mechanisms as well as other relevant phenomena involving the relation between topics. The implementation of opinion models necessarily requires making assumptions to decide which dynamics should be implemented and how. Here, the criterion was to consider only mechanisms that are experimental findings from social psychology and not merely hypothetical assumptions. Additionally, we compared the model's outcomes with 24,035 opinions on controversial issues. These opinions belong to multiple surveys that studied a wide diversity of topics in different countries. As we formulated and numerically integrated the model's master equations, we were able to obtain the model's parameter *k*. This allowed us to replicate the opinions' distribution for every dataset, observe that there are different mechanisms involved in political and nonpolitical discussions, and study which underlying psychological mechanisms could be driving the observed levels of ideological sorting. For instance, for the nonpolitical datasets, *k* was not found to be significantly different from 0, implying that pairwise coherence does not influence these debates. These results are consistent with previous studies that showed that pairwise coherence is characteristic of the political domain.

Moreover, our analysis helped us understand the relationship between the different data sources and the traditional political parties' agendas. For example, the ANES' results, which cover the most relevant and discussed topics within American politics, exhibit a higher proportion of coherent respondents. On the other end, the least aligned political topics are related to the COVID-19 pandemic perception (58). On the one hand, opinions regarding the pandemic are recent and they are not expected, a priori, to be aligned with a particular political platform. On the other hand, the required response to control the virus' spread is not merely political, as it involves both political and personal aspects of every citizen. Also, we know that the perception and compliance with the recommended behavior varied widely across different countries and contexts (59–62).

The simplicity of the proposed model comes with its own limitations. Firstly, we focused our study on controversial issues that exhibit a low proportion of undecided opinions. Whether and how the proposed model could explain other opinions' distributions remains unexplored. Secondly, the model assumes that each agent holds an opinion on two topics. Extending the model to more topics has yet to be explored and presents unique challenges. One challenge is defining the incoherent population when the number of topics is odd, as it becomes impossible to have an equal number of views from each political leaning (positive and negative opinions). Additionally, when exploring a higher number of topics, there will always be two states of coherent agents (all positive or all negative opinions). In contrast, the incoherent ones can be represented by multiple states, each having an equal number of positive and negative opinions. This complexity should be taken into account when calculating sorting values. Thirdly, although it allows us to capture the temporal dynamics of opinions, it is hard to define an adequate time frame in order to match the model's time evolution with actual observed opinion changes. Finally, we observed that it is not possible to replicate the correlation patterns of political opinions by only considering attractive and repulsive homophilic interactions. But this observation does not necessarily imply that pairwise-coherence favoritism is the one and only mechanism driving ideological alignment. More behavioral experiments are needed to understand the importance of each of the multiple factors associated with ideological sorting.

It has become urgent to find and implement solutions to the worryingly rising political polarization. As issue polarization, outgroup hate and distrust, and political segregation increase, democracy gets weaker. One key element of this herculean mission is to fully understand the underlying social, political, and psychological mechanisms driving polarization. To do so, more combined efforts from experimental approaches, theoretical modeling, and large-scale empirical studies are needed.

## Supplementary Material

pgae263_Supplementary_Data

## Data Availability

All simulation data and code for simulations, figures, and the numeric solutions are available at GitHub (https://github.com/lupipedraza/Attraction-by-ingroup-coherence-explains-the-emergence-of-ideological-sorting). This work analyzes data from the following papers and projects: Zimmerman et al. (36), Freira et al. (58), American National Election Studies (63), and Pew Research Center. (https://www.pewresearch.org/politics/dataset/march-2020/https://www.pewresearch.org/religion/dataset/american-trends-panel-wave-6/)

## Preprints

This manuscript was posted on a preprint: arXiv:2304.12559.

## References

[1] Abramowitz A , Saunders K. 2005. Why can’t we all just get along? The reality of a polarized America. Forum. 3:0000102202154088841076.

[2] Baldassarri D , Gelman A. 2008. Partisans without constraint: political polarization and trends in American public opinion. Am J Sociol. 114:408–446. 10.2139/ssrn.1010098 PMC4056259 24932012

[3] Iyengar S , Lelkes Y, Levendusky M, Malhotra N, Westwood SJ. 2019. The origins and consequences of affective polarization in the United States. Ann Rev Pol Sci. 22:129–146.

[4] Boxell L , Gentzkow M, Shapiro J. 2024. Cross-country trends in affective polarization. Rev Econ Stat. 106:557–565.

[5] Finkel EJ , et al 2020. Political sectarianism in America. Science. 370:533–536. 33122374 10.1126/science.abe1715

[6] Iyengar S , Konitzer T, Tedin K. 2018. The home as a political fortress: family agreement in an era of polarization. J Polit. 80:1326–1338.

[7] Tucker J , et al 2018. Social media, political polarization, and political disinformation: a review of the scientific literature. SSRN Electron J. 10.2139/ssrn.3144139

[8] Vosoughi S , Roy D, Aral S. 2018. The spread of true and false news online. Science. 359:1146–1151. 29590045 10.1126/science.aap9559

[9] Bavel JJV , et al 2020. Using social and behavioural science to support COVID-19 pandemic response. Nat Hum Behav. 4:460–471. 32355299 10.1038/s41562-020-0884-z

[10] Lewandowsky S , Jetter M, Ecker UKH. 2020. Using the president's tweets to understand political diversion in the age of social media. Nat Commun. 11:5764. 33173060 10.1038/s41467-020-19644-6 PMC7655817

[11] Tagliazucchi E , Balenzuela P, Travizano M, Mindlin GB, Mininni PD. 2020. Lessons from being challenged by COVID-19. Chaos Solitons Fractals. 137:109923. 32501375 10.1016/j.chaos.2020.109923 PMC7245296

[12] Hartman R , et al 2022. Interventions to reduce partisan animosity. Nat Hum Behav. 6:1194–1205. 36123534 10.1038/s41562-022-01442-3

[13] Combs A , et al 2023. Reducing political polarization in the United States with a mobile chat platform. Nat Hum Behav. 7:1454–1461. 37604989 10.1038/s41562-023-01655-0

[14] Argyle LP , et al 2023. Leveraging AI for democratic discourse: chat interventions can improve online political conversations at scale. Proc Natl Acad Sci U S A. 120:e2311627120. 37788311 10.1073/pnas.2311627120 PMC10576030

[15] Deffuant G , Neau D, Amblard F, Weisbuch G. 2000. Mixing beliefs among interacting agents. Adv Complex Syst. 03:87–98.

[16] Hegselmann R , Krause U. 2002. Opinion dynamics and bounded confidence models, analysis and simulation. J Artif Soc Soc Simul. 5(3).

[17] Lorenz J . 2007. Continuous opinion dynamics under bounded confidence: a survey. Int J Mod Phys C. 18:1819–1838.

[18] Weisbuch G . 2004. Bounded confidence and social networks. Euro Phys J B. 38:339–343.

[19] Baldassarri D , Bearman P. 2007. Dynamics of political polarization. Am Sociol Rev. 72:784–811.

[20] Flache A , et al 2017. Models of social influence: towards the next frontiers. J Artif Soc Soc Simul. 20:2.

[21] Macy M , Kitts J, Flache A, Benard S. 2003. Polarization in Dynamic Networks: A Hopfield Model of Emergent Structure. In: Breiger R, Carley K, Pattison P, editors. Dynamic social network modeling and analysis. Washington (DC): The National Academies Press. p. 162–173.

[22] Axelrod R . 1997. The dissemination of culture: a model with local convergence and global polarization. J Conf Resolut. 41(2):203–226.

[23] Centola D , González-Avella JC, Eguíluz VM, San Miguel M. 2007. Homophily, cultural drift, and the co-evolution of cultural groups. J Conf Resolut. 51:905–929.

[24] Klemm K , Eguíluz VM, Toral R, Miguel MS. 2003. Role of dimensionality in Axelrod's model for the dissemination of culture. Phys A: Stat Mech Appl. 327:1–5.

[25] Guilbeault D , Becker J, Centola D. 2018. Complex contagions: A decade in review. In: Lehmann S, Ahn Y-Y, editors. Complex spreading phenomena in social systems: Influence and contagion in real-world social networks. Cham: Springer International Publishing. p. 3–25.

[26] Barrera Lemarchand F , Semeshenko V, Navajas J, Balenzuela P. 2020. Polarizing crowds: consensus and bipolarization in a persuasive arguments model. Chaos: Interdiscip J Nonlinear Sci. 30:063141. 10.1063/5.0004504 32611083

[27] Mäs M , Flache A. 2013. Differentiation without distancing. Explaining bi-polarization of opinions without negative influence. PLoS One. 8:e74516. 24312164 10.1371/journal.pone.0074516 PMC3842239

[28] Dandekar P , Goel A, Lee DT. 2013. Biased assimilation, homophily, and the dynamics of polarization. Proc Natl Acad Sci U S A. 110:5791–5796. 23536293 10.1073/pnas.1217220110 PMC3625335

[29] Ramos M , et al 2015. How does public opinion become extreme? Sci Rep. 5:10032. 25989484 10.1038/srep10032 PMC4437297

[30] DiMaggio P , Evans J, Bryson B. 1996. Have American's social attitudes become more polarized? Am J Sociol. 102:690–755.

[31] Mason L . 2015. I disrespectfully agree”: the differential effects of partisan sorting on social and issue polarization. Am J Pol Sci. 59:128–145.

[32] Abramowitz AI , Saunders KL. 2008. Is polarization a myth? Source. 70:542–555.

[33] Fiorina MP , Abrams SA, Pope JC. 2008. Polarization in the American public: misconceptions and misreadings. J Polit. 70:556–560.

[34] Fiorina MP , Abrams SJ. 2008. Political polarization in the American public. Ann Rev Polit Sci. 11:563–588.

[35] Lelkes Y . 2016. Mass polarization: manifestations and measurements. Public Opin Q. 80:392–410.

[36] Zimmerman F , Garbulsky G, Ariely D, Sigman M, Navajas J. 2022. Political coherence and certainty as drivers of interpersonal liking over and above similarity. Sci Adv. 8:eabk1909. 35138900 10.1126/sciadv.abk1909 PMC8827732

[37] Abrams D , Marques J, Bown N, Dougill M. 2002. Anti-norm and pro-norm deviance in the bank and on the campus: two experiments on subjective group dynamics. Group Process Intergroup Relat. 5:163–182.

[38] Morrison KR , Miller DT. 2008. Distinguishing between silent and vocal minorities: not all deviants feel marginal. J Pers Soc Psychol. 94:871–882. 18444744 10.1037/0022-3514.94.5.871

[39] Flache A , Macy MW. 2011. Small worlds and cultural polarization. J Math Sociol. 35:146–176.

[40] Flache A , Mäs M. 2008. How to get the timing right. A computational model of the effects of the timing of contacts on team cohesion in demographically diverse teams. Comput Math Organiz Theor. 14:23–51.

[41] Fortunato S , Latora V, Pluchino A, Rapisarda A. 2005. Vector opinion dynamics in a bounded confidence consensus model. Int J Mod Phys C. 16:1535–1551.

[42] Huet S , Deffuant G. 2010. Openness leads to opinion stability and narrowness to volatility. Adv Complex Syst. 13:405–423.

[43] Laguna MF , Abramson G, Zanette DH. 2003. Vector opinion dynamics in a model for social influence. Phys A: Stat Mech Appl. 329:459–472.

[44] Pedraza L , Pinasco JP, Saintier N, Balenzuela P. 2021. An analytical formulation for multidimensional continuous opinion models. Chaos, Solitons & Fractals. 152:111368.

[45] Baumann F , Lorenz-Spreen P, Sokolov IM, Starnini M. 2021. Emergence of polarized ideological opinions in multidimensional topic spaces. Phys Rev X. 11:11012.

[46] Schweighofer S , Garcia D, Schweitzer F. 2020. An agent-based model of multi-dimensional opinion dynamics and opinion alignment. Chaos: Interdiscip J Nonlinear Sci. 30:093139. 10.1063/5.0007523 33003929

[47] Balenzuela P , Pinasco JP, Semeshenko V. 2015. The undecided have the key: interaction-driven opinion dynamics in a three state model. PLoS One. 10:e0139572. 26436421 10.1371/journal.pone.0139572 PMC4593537

[48] Couzin ID , et al 2011. Uninformed individuals promote democratic consensus in animal groups. Science. 334:1578–1580. 22174256 10.1126/science.1210280

[49] Crokidakis N , Anteneodo C. 2012. Role of conviction in nonequilibrium models of opinion formation. Phys Rev E. 86:061127. 10.1103/PhysRevE.86.061127 23367913

[50] Pedraza L , Pinasco JP, Semeshenko V, Balenzuela P. 2023. Mesoscopic analytical approach in a three state opinion model with continuous internal variable. Chaos, Solitons & Fractals. 168:113135.

[51] Shaw A , DeScioli P, Barakzai A, Kurzban R. 2017. Whoever is not with me is against me: the costs of neutrality among friends. J Exp Soc Psychol. 71:96–104.

[52] Mahmoodi A , Bahrami B, Mehring C. 2018. Reciprocity of social influence. Nat Commun. 9:2474. 29946078 10.1038/s41467-018-04925-y PMC6018808

[53] Brady WJ , Wills JA, Jost JT, Tucker JA, Van Bavel JJ. 2017. Emotion shapes the diffusion of moralized content in social networks. Proc Natl Acad Sci U S A. 114:7313–7318. 28652356 10.1073/pnas.1618923114 PMC5514704

[54] Byrne D , Nelson D. 1965. Attraction as a linear function of proportion of positive reinforcements. J Pers Soc Psychol. 1:659–663. 14300244 10.1037/h0022073

[55] Goldenberg A , et al 2022. Homophily and acrophily as drivers of political segregation. Nat Hum Behav. 7:219–230. 36411346 10.1038/s41562-022-01474-9

[56] Bail CA , et al 2018. Exposure to opposing views on social media can increase political polarization. Proc Natl Acad Sci U S A. 115:9216–9221. 30154168 10.1073/pnas.1804840115 PMC6140520

[57] Lorenz J . 2008. Fostering consensus in multidimensional continuous opinion dynamics under bounded confidence. In: Helbing D, editors. Managing complexity: insights, concepts, applications, understanding Complex systems. Berlin Heidelberg: Springer. p. 321–334.

[58] Freira L , Sartorio M, Boruchowicz C, Lopez Boo F, Navajas J. 2021. The interplay between partisanship, forecasted COVID-19 deaths, and support for preventive policies. Humanit Soc Sci Commun. 8:1–10.38617731

[59] Aruguete N , et al 2021. Partisan cues and perceived risks: the effect of partisan social media frames during the COVID-19 crisis in Mexico. J Elect Public Opin Parties. 31:82–95.

[60] Aruguete N , Calvo E, Ventura T. 2021. News sharing, gatekeeping, and polarization: a study of the #Bolsonaro election. Digital J. 9:1–23.

[61] Navajas J , et al 2019. Moral responses to the COVID-19 crisis. R Soc Open Sci. 8:210096. 10.1098/rsos.210096 PMC8439416 34527267

[62] Pavlović T , et al 2022. Predicting attitudinal and behavioral responses to COVID-19 pandemic using machine learning. PNAS Nexus. 1:pgac093. 35990802 10.1093/pnasnexus/pgac093 PMC9381137

[63] American National Election Studies . 2021. ANES 2020 Time Series Study Full Release [dataset and documentation]. February 10, 2022 version. www.electionstudies.org.

