# Supplementary material for: Attraction by pairwise coherence explains the emergence of ideological sorting
Source: PNAS Nexus. 2024 Jul 8;3(7):pgae263. doi: 10.1093/pnasnexus/pgae263 (PMC11288373; doi:10.1093/pnasnexus/pgae263)
Supplement: pgae263_Supplementary_Data [file pgae263_supplementary_data.docx]

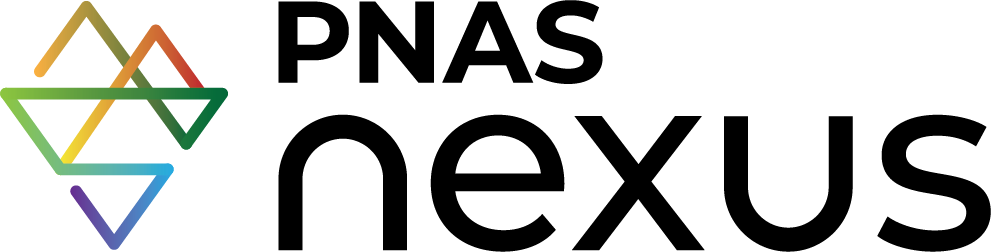


**Supplementary Material for**

Attraction by pairwise coherence explains the emergence of ideological sorting

Federico Zimmerman ^a,b,c,d,e,f,1,^*, Lucía Pedraza ^d,g,1^, Joaquín Navajas ^a,b,c^, Pablo Balenzuela ^d,g^

a. Laboratorio de Neurociencia, Universidad Torcuato Di Tella, Buenos Aires, Argentina

b. Consejo Nacional de Investigaciones Científicas y Técnicas (CONICET), Buenos Aires, Argentina

c. Escuela de Negocios, Universidad Torcuato Di Tella, Buenos Aires, Argentina
d. Universidad de Buenos Aires, Facultad de Ciencias Exactas y Naturales, Departamento de Física, Ciudad Universitaria, Buenos Aires, Argentina.

e. Harvard University, Harvard Business School, Boston, USA

f. Harvard University, Digital, Data and Design Institute, Cambridge, USA

g. CONICET - Instituto de Física Interdisciplinaria y Aplicada (INFINA), Ciudad Universitaria, Buenos Aires, Argentina.

1. F.Z and L.P. contributed equally to this work.

* To whom correspondence may be addressed. **Email**: [fzimmerman@hbs.edu](mailto:fzimmerman@hbs.edu)

**Competing Interest Statement:** The authors declare no competing interest.

**Classification:** Social and Political Sciences, Social Sciences

**Keywords:** political polarization, opinion dynamics, agent-based models, political psychology

**This Word file includes:**

Appendix

**APPENDIX**

**Agents’ possible opinions**

We investigated additional variations of the model, specifically a scenario in which an agent can hold five different opinions per topic: strongly in favor (+2), somewhat in favor (+1), indifferent (0), somewhat against (-1), and strongly against (-2). Given that each agent holds an opinion on two topics, this results in each agent having 25 possible states (Fig. S1A). We considered agents as coherent if their opinions are either both positive or both negative. Conversely, agents are considered incoherent if they hold one positive and one negative opinion. To determine the final states of the model, we conducted simulations varying k in steps of 0.1. Fig. S1B shows the final proportions of coherent and incoherent agents as a function of k. The behavior of this model is consistent with that observed in the simpler three-opinion model: at k=0, the proportions of coherent and incoherent agents are equal, and, while k<1, as k increases, the proportion of coherent agents rises.

**
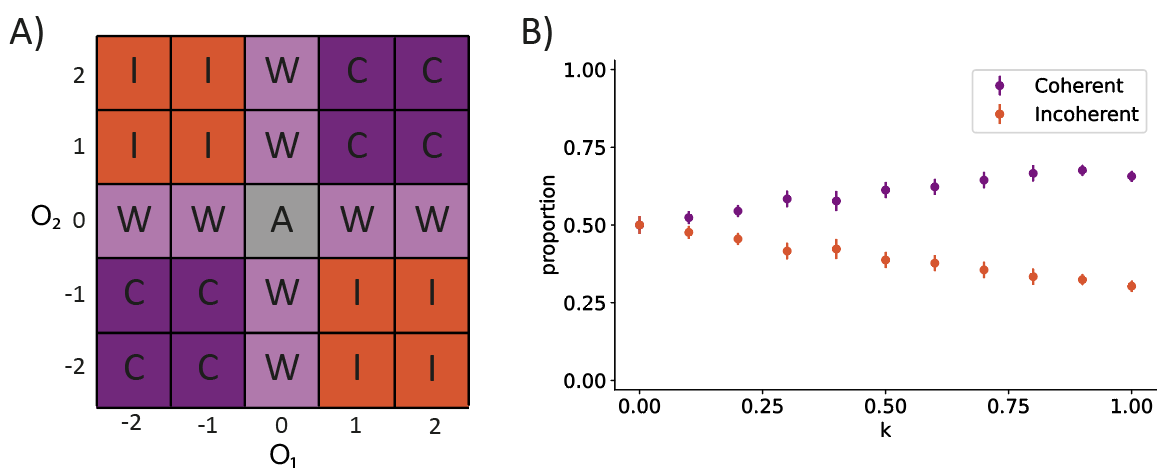
Figure S1: Five-opinion model**. **A)** Each agent holds an independent opinion on two different topics and according to their opinions they are classified in four different communities: coherent (C), incoherent (I), weak (W), or apathetic (A). **B)** The figure depicts the mean (dots) and the standard deviation (error bar) values of the final populations’ proportions in the five-opinion model. The coherent community is shown in purple and the incoherent one in orange.

**Attraction-similarity boundary**

We explored further variations of the model, focusing on the attraction-similarity threshold (T). This threshold determines the degree of similarity at which agents either attract or repel each other. Agents are attracted to each other if their similarity is equal to or exceeds T; otherwise, they repel each other. Given that agents express bidimensional discrete opinions, the distance between any two agents can only take values from the set {0, ¼, ½, ¾, 1}. We did not explore the extreme cases 0 and 1 where agents either always attract or only identical opinions attract. Therefore, we focused on intermediate thresholds of ¼, ½, and ¾. Fig. S2A illustrates these thresholds, showing for each scenario the opinions that attract (shown in orange) and repel (gray) each other for an agent holding two negative opinions (purple dot).

We derived the corresponding equations for each threshold and performed numerical integration to explore their dynamics. Fig. S2B shows the final population distributions for different values of k. Notably, at lower values of T (½ and ¼), the final states are concentrated either in the apathetic or in the coherent populations, a scenario that is not observed in actual opinion data.

Moreover, to test the robustness of our findings, we considered an alternative metric for defining similarity. Instead of the Manhattan distance, we considered the L-infinity distance, which measures the maximum absolute difference between the components of two vectors. We set the threshold at ½, creating the conditions illustrated in Fig. S2A. We observed that in the final state, for k<1, all communities vanish, and there are only coherent and incoherent individuals (Fig. S2B). In this scenario, all points where there are coherent and incoherent individuals represent fixed points according to our model equations.

**
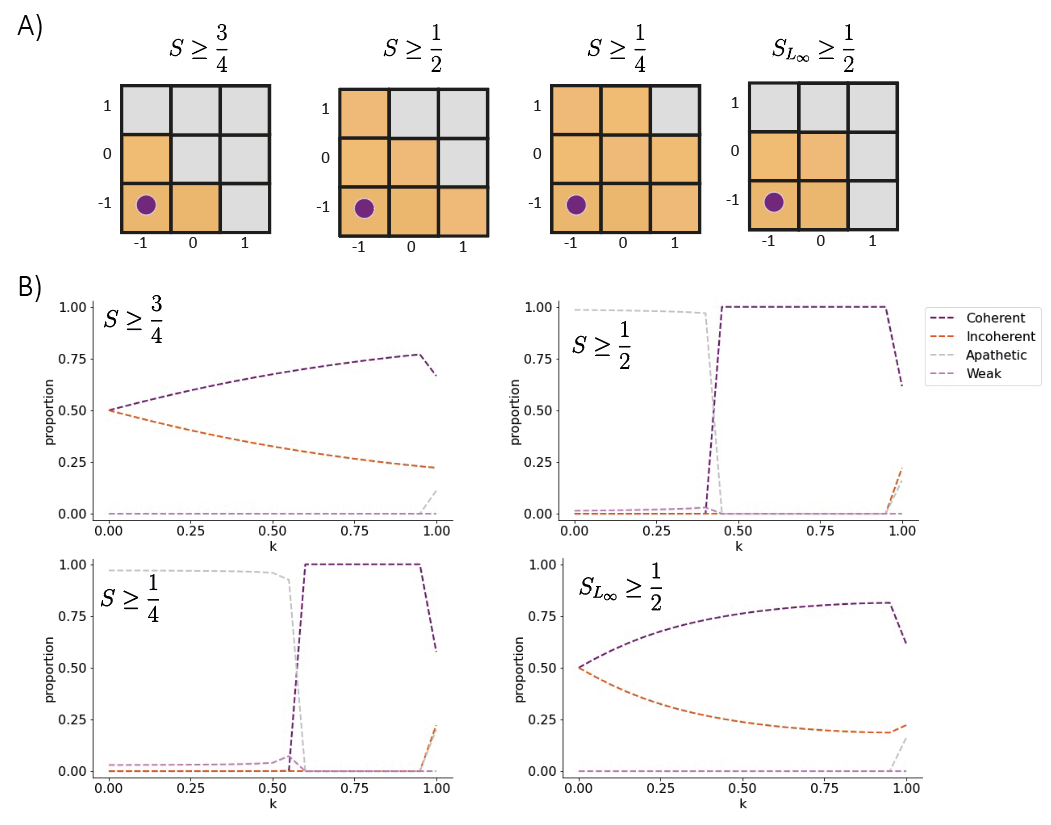
Figure S2**: We explored different values for the attraction-similarity boundary, T. **A)** The figure shows the states that are attractive (in yellow) and those that generate rejection (in gray) for an agent with opinion (-1, -1), as marked in purple, for each value of T. **B)** The figure depicts the numerical solution for each model variant. It shows the final proportion of coherent agents in purple, the final proportion of incoherent agents in orange, the final proportion of apathetic agents in gray, and the final proportion of weak agents in pink.

**Simulations**

We present the results of the model’s simulations. All simulations were done for systems with N=1000 agents and we ran 100 simulations per set of parameters. The parameter k was varied from 0 to 1 by steps of 0.05. Simulations stopped when the system reached the stationary state, and no agent could move from one community to another. We observed the proportion of agents in each community over time and focused on the system’s final state. For every k<1, weak and apathetic communities disappear. Fig. S3 shows the final distribution of coherent and incoherent communities for different k values, over the results obtained from the master equations. This visualization clearly illustrates the correspondence between the two sets of results. When k=0 the proportion of coherent and incoherent agents are the same (C(t_f_ )=I(t_f_ )=0.5) and there is no correlation between opinions. As k increases, so does the proportion of coherent agents. In the specific case where interactions are solely driven by pairwise coherence (k=1), apathetic and incoherent agents do not interact with any other population, so these groups do not change over time.

**
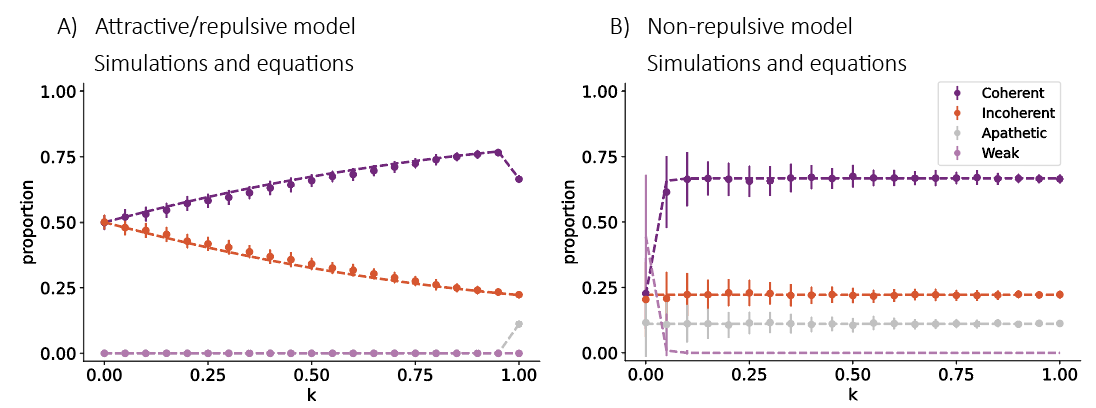
Figure S3**: **Final states.** The model’s final states are shown for different values of k. The figure depicts the mean (dots) and the standard deviation (error bars) values of the final populations’ proportions in the simulations and the numeric solution of the equations (lines). The coherent community is shown in purple, the incoherent one in orange, the apathetic community in gray, and the weak one in pink. **A)** The final states of the attractive-repulsive model. **B)** The final states of the non-repulsive model.

**Online survey**

The online survey had two goals. First, it aimed to determine if there is a unified criterion for identifying whether various issues are considered "political." Secondly, the survey attempted to examine whether the extent to which issues are perceived as political correlates with a model-based measure of ideological sorting.

Participants: The study received approval from the ethics committee for scientific and technological research at the Universidad Abierta Interamericana prior to data collection (protocol number 0-1104). As preregistered (https://aspredicted.org/wp2df.pdf), we targeted 100 participants, balanced in terms of gender, recruited through Prolific. We anticipated capturing a medium-to-large effect size. Thus, a sample of 100 participants would achieve a power of over 0.90 with an alpha level of 0.05. Our sample included 100 U.S. citizens (50 male and 50 female, mean age: 38.8 years, s.d.: 12.6 years). Each participant provided informed consent and correctly answered two attention checks throughout the study.

Stimuli: We considered all groups of statements from the studies included in this work (see Table 1). Notably, Zimmerman et al. 2022 conducted multiple surveys, providing us with three distinct groups of statements: two related to politics and one unrelated to politics. This resulted in a total of eight groups of statements. The number of statements within each group ranged from 5 to 28. To unify the presented stimuli, we randomly selected five statements from each group. Additionally, some datasets contained very similar statements; in such cases, we presented only one of them, randomly selected. This selection process was applied to both the political online surveys by Zimmerman et al. 2022 and the ANES surveys from 2016 and 2020. So, for example, each participant saw five statements from either the ANES 2016 or the ANES 2020. In summary, each participant was presented with six datasets, each containing five statements.

Measures: For each dataset, participants were asked to evaluate each set as a whole. They were asked: "To what extent do you consider these statements to be related to politics?" Responses were collected on a 7-point Likert scale, ranging from "Not at all related" to "Extremely related".

Results: As preregistered, we conducted two analyses. First, we performed a linear mixed-effects model analysis with the dependent variable being the perceived relation to politics. The model included a fixed effect for a dummy variable indicating whether the dataset has been considered related to politics, and participants' IDs as a random effect. Participants rated the datasets that had been considered to be related to politics higher (M=5.66±0.08) compared to the non-political ones (M=1.30±0.06; linear mixed-effects model: b=4.4±0.1, t(499)=39.7, p=2x10^-156^). Secondly, we performed a Spearman correlation analysis between the mean ratings per dataset and the mean k-values obtained from the model, revealing a significant correlation (Spearman correlation: r=0.62, p=0.03; see section ‘Datasets for sorting’ in the appendix for details).

**Datasets for sorting**

Here we explain how we obtained the 16 datasets shown in Fig. 3A from the six studies listed in Table 1. For each ANES survey (2016 and 2020), we created two datasets to ensure that our results were not influenced by the classification method. One dataset included all questions that capture participants' opinions on specific topics (all opinion questions, see Table S1), while the other dataset includes only those questions offering three response options (ternary choice questions). Moreover, because sorting values are calculated based on the proportion of participants expressing coherent opinions across pairs of topics, it was crucial to ensure that these datasets included responses from the same participants on the same issues. From Freira et al. 2021, we obtained four datasets, corresponding to surveys conducted with participants in Argentina, Brazil, Uruguay, and the USA. Furthermore, Zimmerman et al. 2022 conducted four online surveys on political and non-political topics (online surveys, political and non-political), which allowed them to select the five political issues and the five non-political topics they used in an experiment (live experiment, political and non-political). Table S1 displays the most relevant characteristics of these 16 datasets.

| **Number** | **Source and year** | **Set of questions** | **N. of issues** | **N. of part.** | **Country** | **Topic** |
| --- | --- | --- | --- | --- | --- | --- |
| D1 | **Zimmerman et al. 2022** | Online survey. Non-political issues A. | 4 | 88 | Argentina | Non-Political |
| D2 | **Pew Research 2014** | Non-political issues | 5 | 3,278 | USA | Non-Political |
| D3 | **Zimmerman et al. 2022** | Live experiment. Non-political issues. | 5 | 2,406 | Argentina | Non-Political |
| D4 | **Zimmerman et al. 2022** | Online survey. Non-political issues B. | 5 | 92 | Argentina | Non-Political |
| D5 | **Freira et al. 2021** | Uruguayan survey | 8 | 371 | Uruguay | Political |
| D6 | **Freira et al. 2021** | American survey | 7 | 614 | USA | Political |
| D7 | **Zimmerman et al. 2022** | Live experiment. Political issues. | 5 | 2,632 | Argentina | Political |
| D8 | **Freira et al. 2021** | Argentinian survey | 8 | 639 | Argentina | Political |
| D9 | **Zimmerman et al. 2022** | Online survey. Political issues A. | 6 | 88 | Argentina | Political |
| D10 | **ANES 2016** | Ternary choice questions | 11 | 4,270 | USA | Political |
| D11 | **Zimmerman et al. 2022** | Online survey. Political issues B. | 6 | 92 | Argentina | Political |
| D12 | **ANES 2016** | All opinion questions | 28 | 4,270 | USA | Political |
| D13 | **Pew Research 2020** | All opinion questions | 5 | 1,013 | USA | Political |
| D14 | **Freira et al. 2021** | Brazilian survey | 8 | 352 | Brazil | Political |
| D15 | **ANES 2020** | Ternary choice questions | 5 | 8,280 | USA | Political |
| D16 | **ANES 2020** | All opinion questions | 14 | 8,280 | USA | Political |

**Table S1**: The most relevant characteristics of all the datasets used in this work: the source, the set of questions, the year in which the survey was conducted, the number of selected issues, the number of participants who completed each survey, the country, and whether the topics were considered related to politics or not.

**Overview of all the analyzed questions**

**Zimmerman et al. 2022 - Live experiment. Political issues**

1. High school students should be allowed to go on strike.

2. The government should mandate a transgender hiring quota for public servants.

3. The government should subsidize the broadcasting of Argentine football matches.

4. National universities should start charging a fee to those who can afford it.

5. Argentina should sign bilateral trade agreements with the United States.

**Zimmerman et al. 2022- Online survey. Political issues A**

1. In order to progress, Argentina should imitate developed countries.

2. The government should mandate a transgender hiring quota for public servants.

3. No union claim should interfere with the free movement of persons.
4. The government should subsidize the broadcasting of Argentine football matches.

5. The minimum age of criminal responsibility in Argentina (now 16 years) should be reduced.
6. The sentence for rapists should be the death penalty.

Not considered because they were not found to be controversial:

- The state should not subsidize any religious institution.

- The consumption or use of recreational drugs should not be a crime.

**Zimmerman et al. 2022 - Online survey. Political issues B**

1. If someone does graffiti on a public building, they should be detained.
2. Argentina should sign bilateral trade agreements with the United States.

3. National universities should start charging a fee to those who can afford it.

4. High school students should be allowed to go on strike.

5. People with criminal records should not receive any state subsidy.

6. Foreigners should pay for medical care in an Argentine public hospital.

Not considered because they were not found to be controversial:

- Heterosexual couples should have higher priority than gay couples to adopt.
- Abortion should not be a crime.

**Zimmerman et al. 2022 - Live experiment. Non-political issues**

1. One should choose to adopt a dog over a cat.

2. Everyone should use a bidet if they have the possibility.
3. All good barbecues must have blood sausages.
4. Baked schnitzels taste better than fried schnitzels.
5. It is better to go on holidays to the mountains rather than to the seaside.

**Zimmerman et al. 2022 - Online survey. Non-political issues A**

1. Everyone should use a bidet if they have the possibility.

2. When ordering a dozen 'medialunas' more than half should be of animal grease instead of butter.

3. In order to improve the taste, 'empanadas' should carry raisins.

4. One should choose to adopt a dog over a cat.

Not considered because they were not found to be controversial:

- Argentinian Rock radio stations should play more Redondos' songs than Soda Stereo's.

- Whenever possible one should choose the aisle seat over the window seat.

**Zimmerman et al. 2022 - Online survey. Non-political issues B**

1. All good barbecues must have blood sausages.

2. Baked schnitzels taste better than fried schnitzels.
3. It is better to go on holidays to the mountains rather than to the seaside.

4. Maradona should receive the award as 'best history Argentinian soccer player' and Messi should not.

5. ‘Queso y dulce' dessert should have sweet potato and not quince.

Not considered because they were not found to be controversial:

- 'Mate' should be taken with sugar and not bitter.

**ANES 2020 - All opinion questions**

1. V201252 MEDICAL INSURANCE

Where would you place yourself on this scale, or haven’t you thought much about this? From Government insurance plan to Private insurance plan

2. V201258 GOV ASSISTANCE TO BLACKS

Where would you place yourself on this scale, or haven’t you thought much about this? From Government should help blacks to Blacks should help themselves

3. V201306 FEDERAL BUDGET SPENDING: TIGHTENING BORDER SECURITY

What about tightening border security to prevent illegal immigration? Should federal spending on tightening border security to prevent illegal immigration be increased, decreased, or kept the same?

4. V201309 FEDERAL BUDGET SPENDING: DEALING WITH CRIME

What about dealing with crime? Should federal spending on dealing with crime be increased, decreased, or kept the same

5. V201318 FEDERAL BUDGET SPENDING: AID TO THE POOR

What about aid to the poor? Should federal spending on aid to the poor be increased, decreased, or kept the same?

6. V201336 STD ABORTION

There has been some discussion about abortion during recent years. Which one of the opinions on this page best agrees with your view?

a) By law, abortion should never be permitted

b) The law should permit abortion only in case of rape, incest, or when the woman’s life is in danger

c) The law should permit abortion other than for rape/incest/danger to woman but only after need clearly established

d) By law, a woman should always be able to obtain an abortion as a matter of personal choice

7. V201406 SERVICES TO SAME SEX COUPLES

Do you think business owners who provide wedding-related services should be allowed to refuse services to same-sex couples if same-sex marriage violates their religious beliefs, or do you think business owners should be required to provide services regardless of a couple’s sexual orientation?

8. V201409 TRANSGENDER POLICY

Should transgender people - that is, people who identify themselves as the sex or gender different from the one they were born as - have to use the bathrooms of the gender they were born as, or should they be allowed to use the bathrooms of their identified gender?

9. V201415 GAY AND LESBIAN COUPLES BE ALLOWED TO ADOPT

Do you think gay or lesbian couples should be legally permitted to adopt children?

10. V201416 POSITION ON GAY MARRIAGE

Which comes closest to your view?

a) Gay and lesbian couples should be allowed to legally marry
b) Gay and lesbian couples should be allowed to form civil unions but not legally marry

c) There should be no legal recognition of gay or lesbian couples’ relationship

11. V201417 US GOVERNMENT POLICY TOWARD UNAUTHORIZED IMMIGRANTS

Which comes closest to your view about what government policy should be toward unauthorized immigrants now living in the United States?

a) Make all unauthorized immigrants felons and send them back to their home country

b) Have a guest worker program that allows unauthorized immigrants to remain in US to work but only for limited time

c) Allow unauthorized immigrants to remain in US & eventually qualify for citizenship but only if they meet requirements

d) Allow unauthorized immigrants to remain in US & eventually qualify for citizenship without penalties

12. V201418 FAVOR OR OPPOSE ENDING BIRTHRIGHT CITIZENSHIP

Some people have proposed that the U.S. Constitution should be changed so that the children of unauthorized immigrants do not automatically get citizenship if they are born in this country. Do you favor, oppose, or neither favor nor oppose this proposal?

13. V201424 FAVOR OR OPPOSE BUILDING A WALL ON BORDER WITH MEXICO

Do you favor, oppose, or neither favor nor oppose building a wall on the U.S. border with Mexico?

14. V201429 BEST WAY TO DEAL WITH URBAN UNREST

What is the best way to deal with the problem of urban unrest and rioting? Some say it is more important to use all available force to maintain law and order, no matter what results. Others say it is more important to correct the problems of racism and police violence that give rise to the disturbances. And, of course, other people have opinions in between. From Solve problems of racism and police violence to Use all available force to maintain law and order

Not considered because they were not found to be controversial:

- V201300 FEDERAL BUDGET SPENDING: SOCIAL SECURITY

What about Social Security? Should federal spending on Social Security be increased, decreased, or kept the same?

- V201401 GOVERNMENT ACTION ABOUT RISING TEMPERATURES

Do you think the federal government should be doing more about rising temperatures, should be doing less, or is it currently doing the right amount?

- V201412 LAWS PROTECT GAYS/LESBIANS AGAINST JOB DISCRIMINATION

Do you favor or oppose laws to protect gays and lesbians against job discrimination?

**ANES 2020 - Ternary choice questions**

1. V201406 SERVICES TO SAME SEX COUPLES

Do you think business owners who provide wedding-related services should be allowed to refuse services to same-sex couples if same-sex marriage violates their religious beliefs, or do you think business owners should be required to provide services regardless of a couple’s sexual orientation?

2. V201409 TRANSGENDER POLICY

Should transgender people - that is, people who identify themselves as the sex or gender different from the one they were born as - have to use the bathrooms of the gender they were born as, or should they be allowed to use the bathrooms of their identified gender?

3. V201424 FAVOR OR OPPOSE BUILDING A WALL ON BORDER WITH MEXICO

Do you favor, oppose, or neither favor nor oppose building a wall on the U.S. border with Mexico?

4. V201418 FAVOR OR OPPOSE ENDING BIRTHRIGHT CITIZENSHIP

Some people have proposed that the U.S. Constitution should be changed so that the children of unauthorized immigrants do not automatically get citizenship if they are born in this country. Do you favor, oppose, or neither favor nor oppose this proposal?

5. V201415 GAY AND LESBIAN COUPLES BE ALLOWED TO ADOPT

Do you think gay or lesbian couples should be legally permitted to adopt children?

**ANES 2016 - All opinion questions**

1. V161113 Healthcare

Do you favor, oppose, or neither favor nor oppose the health care reform law passed in 2010? This law requires all Americans to buy health insurance and requires health insurance companies to accept everyone.

2. V161184 Insurance

Where would you place yourself on this scale, or haven’t you thought much about this? From Government insurance plan to Private insurance plan.

3. V161196 Wall

Do you favor, oppose, or neither favor nor oppose building a wall on the U.S. border with Mexico?

4. V161198 Black

Where would you place yourself on this scale, or haven’t you thought much about this? From Government should help Blacks to Blacks should help themselves.

5. V161201 Environment

Where would you place yourself on this scale, or haven’t you thought much about this? From Regulate business to protect the environment and create jobs to No regulation because it will not work and will cost jobs.

6. V161214 Syrian

Do you favor, oppose, or neither favor nor oppose allowing Syrian refugees to come to the United States?

7. V161228 TransBathroom

Should transgender people – that is, people who identify themselves as the sex or gender different from the one they were born as – have to use the bathrooms of the gender they were born as, or should they be allowed to use the bathrooms of their identified gender?

8. V161227 Same Sex Service

Do you think business owners who provide wedding-related services should be allowed to refuse services to same-sex couples if same-sex marriage violates their religious beliefs, or do you think business owners should be required to provide services regardless of a couple’s sexual orientation?

9. V161193 Birthright

Some people have proposed that the U.S. Constitution should be changed so that the children of unauthorized immigrants do not automatically get citizenship if they are born in this country. Do you favor, oppose, or neither favor nor oppose this proposal?

10. V161204 Affirmative Action

Do you favor, oppose, or neither favor nor oppose allowing universities to increase the number of black students studying at their schools by considering race along with other factors when choosing students?

11. V161213 ISIS

Do you favor, oppose, or neither favor nor oppose the U.S. sending ground troops to fight Islamic militants, such as ISIS, in Iraq and Syria?

12. V161229 Gay protection

Do you favor or oppose laws to protect gays and lesbians against job discrimination?

13. V161232 Abortion

There has been some discussion about abortion during recent years. Which one of the opinions on this page best agrees with your view?

a) By law, abortion should never be permitted.

b) By law, only in case of rape, incest, or woman’s life in danger.

c) By law, for reasons other than rape, incest, or woman’s life in danger if need established.

d) By law, abortion as a matter of personal choice.

14. V161233 death penalty

Do you favor or oppose the death penalty for persons convicted of murder?

15. V161343 protesters

When protestors get ‘roughed up’ for disrupting political events, how much do they generally deserve what happens to them?

16. V161346 feminism

How well does the term feminist’ describe you?

17. V162123 Countries like America

‘The world would be a better place if people from other countries were more like Americans.’ Do you [agree strongly, agree somewhat, neither agree nor disagree, disagree somewhat, or disagree strongly / disagree strongly, disagree somewhat, neither agree nor disagree, agree somewhat, or agree strongly] with this statement?

18. V162169 forefathers

‘Our country would be great if we honor the ways of our forefathers, do what the authorities tell us to do, and get rid of the ‘rotten apples’ who are ruining everything.’ (Do you [agree strongly, agree somewhat, neither agree nor disagree, disagree somewhat, or disagree strongly / disagree strongly, disagree somewhat, neither agree nor disagree, agree somewhat, or agree strongly] with this statement?)

19. V162170 strong leader

What our country really needs is a strong, determined leader who will crush evil and take us back to our true path.’ (Do you [agree strongly, agree somewhat, neither agree nor disagree, disagree somewhat, or disagree strongly / disagree strongly, disagree somewhat, neither agree nor disagree, agree somewhat, or agree strongly] with this statement?)

20. V162210 traditional family

‘This country would have many fewer problems if there were more emphasis on traditional family ties.’ (Do you [agree strongly, agree somewhat, neither agree nor disagree, disagree somewhat, or disagree strongly / disagree strongly, disagree somewhat, neither agree nor disagree, agree somewhat, or agree strongly] with this statement?)

21. V162211 help blacks

’Irish, Italians, Jewish and many other minorities overcame prejudice and worked their way up. Blacks should do the same without any special favors.’ Do you [agree strongly, agree somewhat, neither agree nor disagree, disagree somewhat, or disagree strongly / disagree strongly, disagree somewhat, neither agree nor disagree, agree somewhat, or agree strongly] with this statement?

22. V162221 hispanics

How important is it that more Hispanics be elected to political office?

23. V162244 equality

‘This country would be better off if we worried less about how equal people are.’ (Do you [agree strongly, agree somewhat, neither agree nor disagree, disagree somewhat, or disagree strongly / disagree strongly, disagree somewhat, neither agree nor disagree, agree somewhat, or agree strongly] with this statement?)

24. V162266 traditions

Now thinking about minorities in the United States. Do you [agree strongly, agree somewhat, neither agree nor disagree, disagree somewhat, or disagree strongly / disagree strongly, disagree somewhat, neither agree nor disagree, agree somewhat or agree strongly] with the following statement? ‘Minorities should adapt to the customs and traditions of the United States’

25. V162268 immigrants economy

And now thinking specifically about immigrants. (Do you [agree strongly, agree somewhat, neither agree nor disagree, disagree somewhat, or disagree strongly /disagree strongly, disagree somewhat, neither agree nor disagree, agree somewhat or agree strongly] with the following statement?) ‘Immigrants are generally good for America’s economy.’

26. V162270 immigrants crime

(Do you [agree strongly, agree somewhat, neither agree nor disagree, disagree somewhat, or disagree strongly /disagree strongly, disagree somewhat, neither agree nor disagree, agree somewhat or agree strongly] with the following statement?) ‘Immigrants increase crime rates in the United States.’

27. V162276 differences

Please say to what extend you agree or disagree with the following statement: ‘The government should take measures to reduce differences in income levels’. (Do you [agree strongly, agree somewhat, neither agree nor disagree, disagree somewhat, or disagree strongly / disagree strongly, disagree somewhat, neither agree nor disagree, agree somewhat or agree strongly]?)

28. V162295 torture

Do you favor, oppose, or neither favor nor oppose the U.S. government torturing people who are suspected of being terrorists, to try to get information?

Not considered because they were not found to be controversial:

- V161154 Military

How willing should the United States be to use military force to solve international problems?

- V161226 Parental leave

Do you favor/oppose, or neither favor nor oppose requiring employers to offer paid leave to parents of new children?

- V162125x Flag
How good/bad does R feel to see American flag?

- V162150x equal pay
Favor/oppose equal pay for men and women

- V162168 free thinkers
‘Our country needs free thinkers who will have the courage to defy traditional ways, even if this upsets many people.’ Do you [agree strongly, agree somewhat, neither agree nor disagree, disagree somewhat, or disagree strongly / disagree strongly, disagree somewhat, neither agree nor disagree, agree somewhat, or agree strongly] with this statement?

- V162186 business regulation

How much government regulation of business is good for society?

**ANES 2016 - Ternary choice questions**

1. V161113 Healthcare

Do you favor, oppose, or neither favor nor oppose the health care reform law passed in 2010? This law requires all Americans to buy health insurance and requires health insurance companies to accept everyone.

2. V161196 Wall

Do you favor, oppose, or neither favor nor oppose building a wall on the U.S. border with Mexico?

3. V161214 Syrian

Do you favor, oppose, or neither favor nor oppose allowing Syrian refugees to come to the United States?

4. V161228 TransBathroom

Should transgender people – that is, people who identify themselves as the sex or gender different from the one they were born as – have to use the bathrooms of the gender they were born as, or should they be allowed to use the bathrooms of their identified gender?

5. V161227 Same Sex Service

Do you think business owners who provide wedding-related services should be allowed to refuse services to same-sex couples if same-sex marriage violates their religious beliefs, or do you think business owners should be required to provide services regardless of a couple’s sexual orientation?

6. V161193 Birthright

Some people have proposed that the U.S. Constitution should be changed so that the children of unauthorized immigrants do not automatically get citizenship if they are born in this country. Do you favor, oppose, or neither favor nor oppose this proposal?

7. V161204 Affirmative Action

Do you favor, oppose, or neither favor nor oppose allowing universities to increase the number of black students studying at their schools by considering race along with other factors when choosing students?

8. V161213 ISIS
Do you favor, oppose, or neither favor nor oppose the U.S. sending ground troops to fight Islamic militants, such as ISIS, in Iraq and Syria?

9. V161229 Gay protection

Do you favor or oppose laws to protect gays and lesbians against job discrimination?

10. V161233 death penalty

Do you favor or oppose the death penalty for persons convicted of murder?

11. V162295 torture

Do you favor, oppose, or neither favor nor oppose the U.S. government torturing people who are suspected of being terrorists, to try to get information?

Not considered because they were not found to be controversial:

- V161226 Parental leave

Do you favor/oppose, or neither favor nor oppose requiring employers to offer paid leave to parents of new children?

**Freira et al. 2021 - Argentinian survey**

1. Schools should reopen before the end of the academic year.
2. Non-essential public meetings should be banned until the development of a vaccine.
3. People should be allowed to leave their homes and exercise at least once a day.
4. People over 70 years old should not be allowed to leave their homes until a vaccine is found.
5. People who are found in a public space without a valid reason should have a criminal record.
6. People should be allowed to freely travel within the country without requesting permission from the government.
7. The government should track the movements of all patients who tested positive for COVID-19 using their cell-phone data.
8. The government should fine those individuals who upload false information to the official virus-tracking app.

Not considered because they were not found to be controversial:

- The government should force the citizens to share their geolocation through an official virus-tracking app.

**Freira et al. 2021 - Brazilian survey**

1. Schools should reopen and resume face-to-face teaching by the end of the year.
2. Non-essential public meetings should be banned until the development of a vaccine.
3. Gatherings of more than 10 people should not be allowed until a vaccine is developed.
4. People over 70 years old should not be allowed to leave their homes until a vaccine is found.
5. People who are found in a public space without a valid reason should have a criminal record.
6. The government should track the movements of all patients who tested positive for COVID-19 using their cell-phone data.
7. The government should fine those individuals who upload false information to the official virustracking app.
8. The government should force the citizens to share their geolocation through an official virus-tracking app.

Not considered because they were not found to be controversial:
- All businesses and stores should reopen without requiring them to obtain an official authorization.

**Freira et al. 2021 - Uruguayan survey**

1. Universities should reopen and resume face-to-face teaching by the end of the year.
2. Gatherings of more than 10 people should not be allowed until a vaccine is developed.
3. The government should fine people who do not respect social distance in the street.
4. People diagnosed with COVID19 should have a criminal record if they are found in a public space during the period when transmission risk is high.
5. People should be allowed to freely travel within the country without requesting permission from the government.
6. The government should track the movements of all patients who tested positive for COVID-19 using their cell-phone data.
7. The government should fine those individuals who upload false information to the official virus-tracking app.
8. The government should force the citizens to share their geolocation through an official virus-tracking app.

Not considered because they were not found to be controversial:

- People over 70 years old should not be allowed to leave their homes until a vaccine is found.

**Freira et al. 2021 - American survey**

1. All schools in the United States should reopen before the end of 2020.
2. All non-essential public events should be banned until a vaccine is found.
3. The Federal Government should track the location of people infected with COVID-19 using a mobile phone app.
4. Wearing a mask in public spaces should be optional.
5. People over 70 years old should not be allowed to leave their homes until a vaccine is found.
6. People should request permission to the Federal Government to travel from one state to another.
7. Until a vaccine is found, the Federal Government should not allow mass protests in the United States.

**Pew Research 2020 - All opinion questions**

Is your overall opinion of [INSERT ITEM] very favorable, mostly favorable, mostly unfavorable, or very unfavorable?
1. The Department of Homeland Security

2. The Internal Revenue Service, the IRS

3. The Justice Department

4. The Department of Veterans Affairs, the VA

5. The Immigration and Customs Enforcement, known as ICE [PRONOUNCED: ‘ice’]

Not considered because they were not found to be controversial:

- The Centers for Disease Control and Prevention, the CDC

- The Department of Health and Human Services, the HHS

- The Census Bureau

- The Postal Service

- The Federal Reserve

**Pew Research 2014 - Non-political issues**

1. In the past week, did you donate money, time or goods to help the poor and needy?

2. In the past week, did you tell a white lie?

3. In the past week, did you lose your temper?

4. In the past week, did you ever eat too much?

5. In the past week, did you meditate to cope with stress?
